# A Decision Aid for Patients Considering Surgery for Sciatica: Codesign and User‐Testing With Patients and Clinicians

**DOI:** 10.1111/hex.14111

**Published:** 2024-06-19

**Authors:** Julie Ayre, Richie Kumarage, Hazel Jenkins, Kirsten J. McCaffery, Christopher G. Maher, Mark J. Hancock

**Affiliations:** ^1^ Sydney Health Literacy Lab, Sydney School of Public Health, Faculty of Medicine and Health The University of Sydney Sydney New South Wales Australia; ^2^ Department of Chiropractic, Faculty of Medicine, Health and Human Sciences Macquarie University Macquarie Park New South Wales Australia; ^3^ Institute of Musculoskeletal Health, Faculty of Medicine and Health The University of Sydney and Sydney Local Health District Sydney New South Wales Australia; ^4^ Department of Health Professions, Faculty of Medicine, Health and Human Sciences Macquarie University Macquarie Park New South Wales Australia

**Keywords:** health literacy, patient decision aid, patient education, sciatica, shared decision making, surgery

## Abstract

**Background:**

Surgery can help patients with leg pain caused by sciatica recover faster, but by 12 months outcomes are similar to nonsurgical management. For many the decision to have surgery may require reflection, and patient decision aids are an evidence‐based clinical tool that can help guide patients through this decision.

**Objective:**

The aim of this study was to develop and refine a decision aid for patients with sciatica who are deciding whether to have surgery or ‘wait and see’ (i.e., try nonsurgical management first).

**Design:**

Semistructured interviews with think‐aloud user‐testing protocol.

**Participants:**

Twenty clinicians and 20 patients with lived experience of low back pain or sciatica.

**Outcome Measures:**

Items from Technology Acceptance Model, Preparation for Decision Making Scale and Decision Quality Instrument for Herniated Disc 2.0 (knowledge instrument).

**Methods:**

The prototype integrated relevant research with working group perspectives, decision aid standards and health literacy guidelines. The research team refined the prototype through seven rounds of user‐testing, which involved discussing user‐testing feedback and implementing changes before progressing to the next round.

**Results:**

As a result of working group feedback, the decision aid was divided into sections: before, during and after a visit to the surgeon. Across all rounds of user‐testing, clinicians rated the resource 5.9/7 (SD = 1.0) for perceived usefulness, and 6.0/7 for perceived ease of use (SD = 0.8). Patients reported the decision aid was easy to understand, on average correctly answering 3.4/5 knowledge questions (SD = 1.2) about surgery for sciatica. The grade reading score for the website was 9.0. Patients scored highly on preparation for decision‐making (4.4/5, SD = 0.7), suggesting strong potential to empower patients. Interview feedback showed that patients and clinicians felt the decision aid would encourage question‐asking and help patients reflect on personal values.

**Conclusions:**

Clinicians found the decision aid acceptable, patients found it was easy to understand and both groups felt it would empower patients to actively engage in their care and come to an informed decision that aligned with personal values. Input from the working group and user‐testing was crucial for ensuring that the decision aid met patient and clinician needs.

**Patient or Public Contribution:**

Patients and clinicians contributed to prototype development via the working group.

## 1 Introduction

Shared decision‐making is increasingly recognised as a cornerstone of person‐centred care, and describes the process of patients and clinicians working together to make a health decision that considers the medical evidence about a health issue, as well as the patient's personal values and preferences [1]. This process is particularly important when the evidence does not recommend one treatment over another [2].

The value of shared decision‐making can be illustrated through the example of a patient weighing up whether to have surgery to treat sciatica associated with disc herniation. Sciatica is characterised by pain that radiates from the lower back into the leg, usually below the knee and sometimes into the foot and toes [3]. Though many patients will recover on their own within the first few months, for some the pain persists into the long term [4]. Sciatica can be highly disabling, and a key treatment decision is whether to have surgery to help speed up the recovery or continue with nonsurgical management such as medicine, injections and physical therapy.

For many patients, this decision‐making process can be challenging because the evidence suggests surgery is likely to speed up recovery but will not improve their long‐term outcomes compared to a nonsurgical approach. Systematic reviews have identified only a single randomised‐controlled trial with low risk of bias that has properly investigated this topic [5, 6, 7, 8, 9]. This trial showed that for adults with sciatica, ‘early surgery’ resulted in greater reduction in leg pain compared to prolonged nonsurgical management in the short term and medium term [10]. By 12 months, recovery across both groups was the same. Other studies, though less rigorously evaluated, have reported similar findings [11].

Some have argued that after a few months of trying nonsurgical management options, clinicians should discuss surgery as a treatment option with patients, including its potential benefits and harms [9, 12]. However, discussions alone may not be sufficient, particularly given the complexity of the evidence and a power imbalance between patients and clinicians that may discourage question‐asking [13, 14, 15]. Patient decision aids are a type of tool that can support shared decision‐making by providing balanced, evidence‐based information and guiding patients to think about which benefits and harms are most important to them [16, 17]. They are underpinned by a strong evidence base; a systematic review of over 100 trials of decision aids across various health conditions showed that they effectively increase knowledge, accuracy of risk perceptions and alignment between informed values and health decisions, compared to usual care [17].

A few decision aids for patients considering surgery for sciatica already exist. However, some were developed before the most recent evidence became available [18, 19]. Others, developed for Danish [20] and US contexts [21, 22], were designed for use by surgeons or as a publicly available resource, respectively. Timely, targeted and appropriate access to these decision aids could be improved by involving primary care and allied health clinicians who are already involved in the patients' care and who are therefore well‐placed to help their patients prepare for a surgical appointment ahead of time. For example, in many countries, general practitioners (GPs) are responsible for the referral to the surgeon, who in turn confirms the diagnosis and provides advice about the surgery. Other clinicians such as physiotherapists or chiropractors are also relevant as patients may initially present to these clinicians with symptoms of sciatica. Meeting the needs of patients with sciatica and their clinicians is important as uptake of patient decision aids is typically poor, even for those that meet best‐practice guidelines and demonstrate evidence of significantly improving patient outcomes [23].

This study reports the development and user‐testing of a decision aid for patients considering surgery for sciatica [24, 25]. The decision aid also incorporated health literacy into the design, an identified gap in decision aid research [26] and a novel interactive values clarification activity to help users reflect on personal values, preferences and goals [27, 28].

## 2 Methods

### 2.1 Study Design

The research team first designed an initial prototype for the decision aid. Semistructured interviews including a ‘think‐aloud’ protocol then supported iterative refinement of the prototype. Ethical approval for the study was obtained from the University of Sydney Human Research Ethics Committee (Project number 2022/678).

### 2.2 Prototype Development

#### 2.2.1 Scope

The target patient group for the decision aid was people with sciatica caused by lumbar disc herniation with sciatic leg pain lasting less than 6 months. The decision aid was considered most suitable for patients who clinicians considered appropriate for referral to a spinal surgeon for an opinion on back surgery, or for patients who had already received a referral. It is less suitable for patients where prompt surgery is indicated, for example, those with progressive neurological loss or cauda equina syndrome. Target clinicians were GPs, chiropractors, physiotherapists and surgeons. The key decision under consideration was choosing between surgery or a ‘wait and see’ option (delaying the decision to have surgery to see if the pain resolved with time or nonsurgical management).

#### 2.2.2 Design and Content

Design was informed by the 2021 International Patient Decision Aid Standards (IPDAS) [26, 29, 30, 31] and IPDAS minimum standards for patient decision aids [32]. This approach ensured that the decision aid provided all information needed to make an informed decision and that information was balanced, reliable and transparent. Alignment with these standards was also enhanced by adhering to health literacy guidelines [33, 34]. These guidelines advocate designs that use plain language, break down information into sections and incorporate white space. Use of plain language was supported by the Sydney Health Literacy Lab Health Literacy Editor [35].

Content about treatment options was informed by five systematic reviews summarising the research on this topic, including one published in 2023 [5, 6, 7, 8, 9]. Each review identified a 2007 trial by Peul et al. [10] as the single most relevant, high‐quality study evaluating the effects of surgery compared to nonsurgical management. This study showed that surgery provided greater reduction in leg pain compared to prolonged nonsurgical management in the short term and medium term, but that by 12 months, recovery across both groups was the same. Other lower quality evidence also supported these conclusions [11].

To support reflection on personal values we developed an online browser‐based activity. Patients were instructed to rate the extent that opposing reasons for surgical and nonsurgical management was important to them. As each rating was completed, a dynamic bar graph visually represented the collective weighting of these ratings towards the two treatment options. Once a decision about treatment was made, the user received feedback about whether the decision aligned with their stated values. Research suggests that this format may improve alignment between a person's values and their decision and reduce conflicted feelings compared to standard rating scales [27, 28].

#### 2.2.3 Codesign

To support implementation, a stakeholder working group comprising clinicians (GPs, surgeons, chiropractors, physiotherapists) and people with experience of sciatica or low back pain was established (*N* = 16). Members were engaged through a series of online workshops to clarify the clinical context (e.g., typical workflow), and clinician and patient needs. The research team developed the initial prototype over the course of these workshops.

### 2.3 User‐Testing

#### 2.3.1 Participants and Recruitment

Eligible clinicians were health practitioners registered with the Australian Health Practitioner Regulation Agency and with experience managing patients with sciatica. Clinicians were recruited through health network groups including professional mailing lists and Facebook groups. Eligible patients were Australian adults who had current or previous experience of low back pain or sciatica, with their worst episode being at least a moderate level of interference in their daily activities. Patient participants were recruited via referral from participating clinicians and via social media advertisements (Facebook and Instagram; Appendix S1). Referring clinicians were asked to invite patients with current or previous sciatica or low back pain, and whose pain had at least a moderate impact on their daily activities. Interested participants then completed a brief survey to undergo screening and respond to questions used to support purposive sampling (Appendix S2).

Patients and clinicians were purposively sampled to ensure a range of experiences and demographics including age and gender. For clinicians, we also sought experiences across different professions who may be involved in the patients' care (GP, physiotherapist, chiropractor or surgeon). For patients, we sought to capture perspectives of people with differing levels of education and experiences of sciatica or low back pain. Recruitment took place between December 6, 2022 and September 7, 2023.

#### 2.3.2 Interviews and Iterative Optimisation

Each participant gave feedback via a single semistructured interview over Zoom. Participants shared their screen and went through the clinician user guide (if a clinician) and the decision aid prototype while ‘thinking aloud’ [36]. They received minimal assistance to use the intervention, with neutral prompts to encourage the participant to continue thinking aloud. Audio‐visual data were recorded and transcribed. Interviewers (Authors 1 and 2) made notes and reflections during and after the interviews.

After completing the interview, clinician acceptability was assessed using relevant items from the Technology Acceptance Model (perceived usefulness and perceived ease of use subscales) [37]. Patients completed items from the Preparation for Decision Making Scale [38], a validated assessment of patient perceptions of a decision aid's usefulness, and an adapted version of the Decision Quality Instrument for Herniated Disc 2.0 [39], a validated assessment of knowledge about surgery for sciatica. In this study, the latter assessed gist rather than verbatim understanding of key information available in the decision aid.

After each round of interviews (approximately 5–7 participants), feedback was collated and potential modifications were discussed by the research team. In line with prototype optimisation criteria [40], changes were prioritised if they:

1. supported patient or clinician engagement in shared decision‐making
2. were consistent with the intervention's guiding principles, including the IPDAS minimum standards
3. were uncontroversial and easy to implement
4. were repeated by several participants

Clinician members of the research team had substantial experience managing people with sciatica and identifying people who may need opinion for surgical treatment.

Results summarise the key improvements made to the decision aid. Screenshots, participant quotes and descriptions of user‐testing observations demonstrate support for these changes. The final version of the decision aid can be viewed at https://www.sydneyhealthliteracylab.org.au/decision-aid.

## 3 Results

### 3.1 Prototype Development

**Figure 1:**
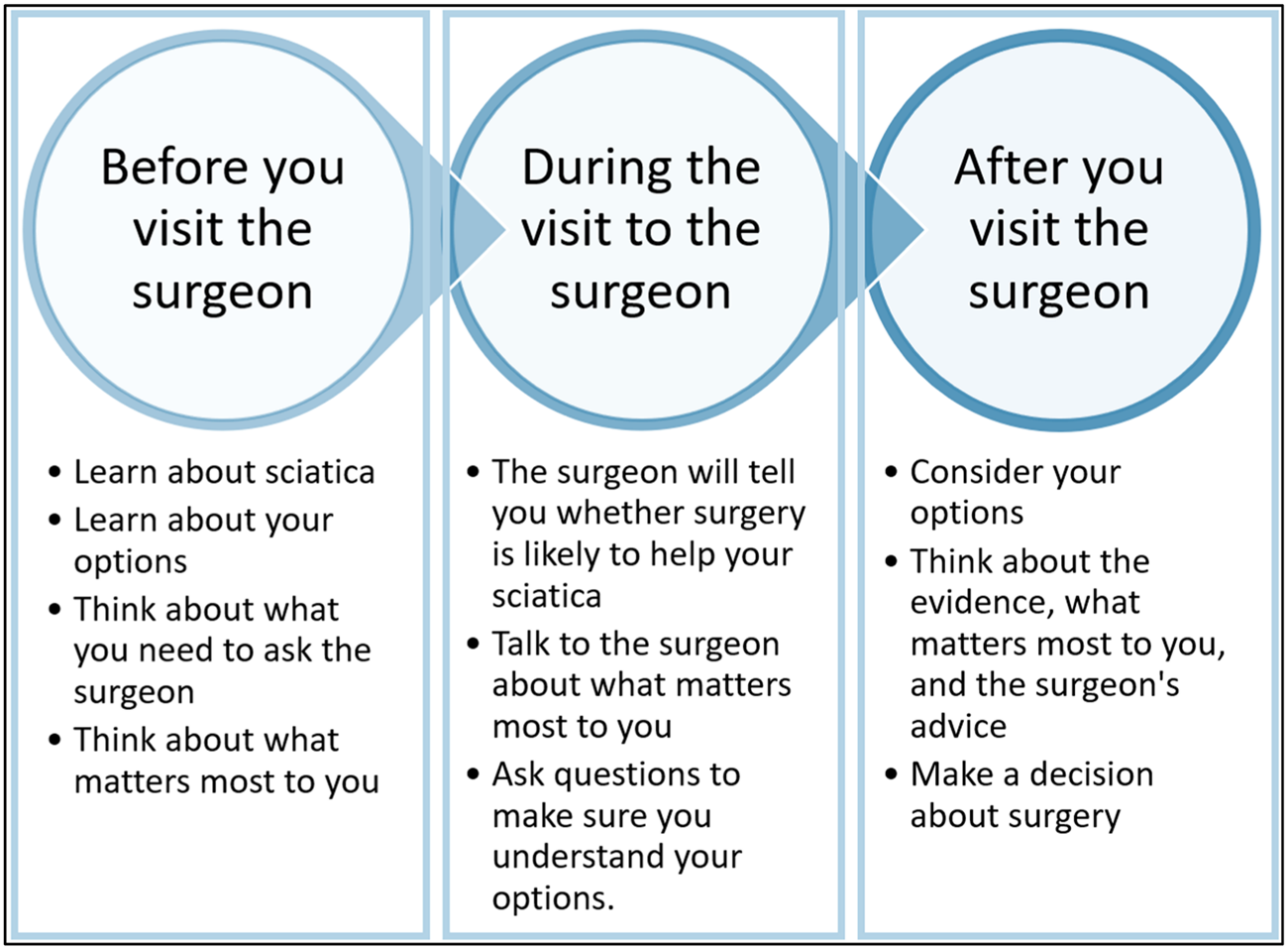
The three sections of the initial patient decision aid prototype.

Feedback from the working group helped refine the initial prototype (Table 1). Most notably, the decision aid was divided into three segments that supported patients before, during and after consultation with a surgeon (Figure 1). This reflects the fact that the surgeon is responsible for confirming the diagnosis and providing advice about whether the patient is likely to be a good candidate for surgery. Preparing patients for the visit also addressed patient concerns that talking to a surgeon and asking them questions could be intimidating and challenging.

**Table 1:** Summary of stakeholder feedback and implications for prototype design.

Issue	Feedback	Design implications and priorities
Scope	Surgeons discussed that the diagnosis of sciatica is typically confirmed by a surgeon rather than a general practitioner (GP) Patients wanted the decision aid to help them prepare before seeing the surgeon, as well as to use during and after the appointment with the surgeon	End‐users
		Patients with a referral to see a surgeon Referring GP, allied health professional involved in the patient's care, or staff at surgical clinic
		Structure
		Prototype divided into three sections: Before, during and after a visit to the surgeon
Mode of delivery	Patient and clinician preference for both print (PDF) and online formats GP and allied health preference for brief user guide to help them understand the decision aid's purpose and how to use it	Structure
		Primarily a PDF (printable) format, that could be easily adapted as online content Brief clinician user guide to explain the purpose of a patient decision aid, its intended use (including which patients it would be best suited to), and further detail about the evidence base
Goal–Patient empowerment	Patients strongly emphasised the importance of features that encourage or ‘give permission’ to ask the surgeon questions Patients wanted the decision aid to reduce/Help them understand jargon Patients wanted to feel less pressure to make a decision quickly Patients wanted to emphasise that you can get a second opinion, and more resources for further reading	Content
		Strongly emphasise the right to ask questions, and to ask the clinician to explain something if the patient does not understand Simple language and glossary of key terms. Initial prototype had a grade reading score of 9.5 as assessed by the Health Literacy Editor, or Grade 8.1 with the two keywords ‘surgery’ and ‘sciatica’ removed Advice that it is OK to take one's time, and to get a second opinion was added to decision aid prototype Links for further reading and relevant research
Goal–Encouraging reflection on personal values	Patients and clinicians expressed a preference for interactive values clarification activities as opposed to static rating scales	Structure/Content
		Interactive online values clarification task providing real‐time visualisation of how a person's preferences relate to the decision

### 3.2 User‐Testing

Out of 89 clinicians and 70 patients who expressed interest in the study, 20 of each were selected to take part in user‐testing (Table 2 and Appendix S5). Interviews took place across seven rounds of user‐testing.

**Table 2:** Participant characteristics.

Clinician characteristics	*N*	%	Patient characteristics	*N*	%
Profession			Age		
Physiotherapist	6	30	18–39	7	35
Chiropractor	5	25	40–49	5	25
General practitioner	6	30	50–59	5	25
Surgeon	4	20	60+	3	15
Gender			Gender		
Male	10	50	Male	8	40
Female	10	50	Female	11	55
Years' experience with low back pain patients			Prefer not to say	1	5
Less than 5 years	3	15	Pain experience (current)		
5–9 years	7	35	Back pain only	6	30
≥10 years	10	50	Back pain and sciatica	12	60
Country of birth			Education		
Australia	11	55	Less than university	11	55
Other	9	45	University	9	45
Setting			Country of birth		
Private	15	75	Australia	11	55
Public	2	10	Other	9	45
Urban	15	75	Total	20	
Regional/Rural	4	25			
Total	20				

Most changes could be categorised as enhancing one of three key aspects: clinician acceptability, ease of understanding and patient empowerment. For each of these categories, we also report relevant survey items completed after the interview.

#### 3.2.1 Clinician Acceptability

**Figure 2:**
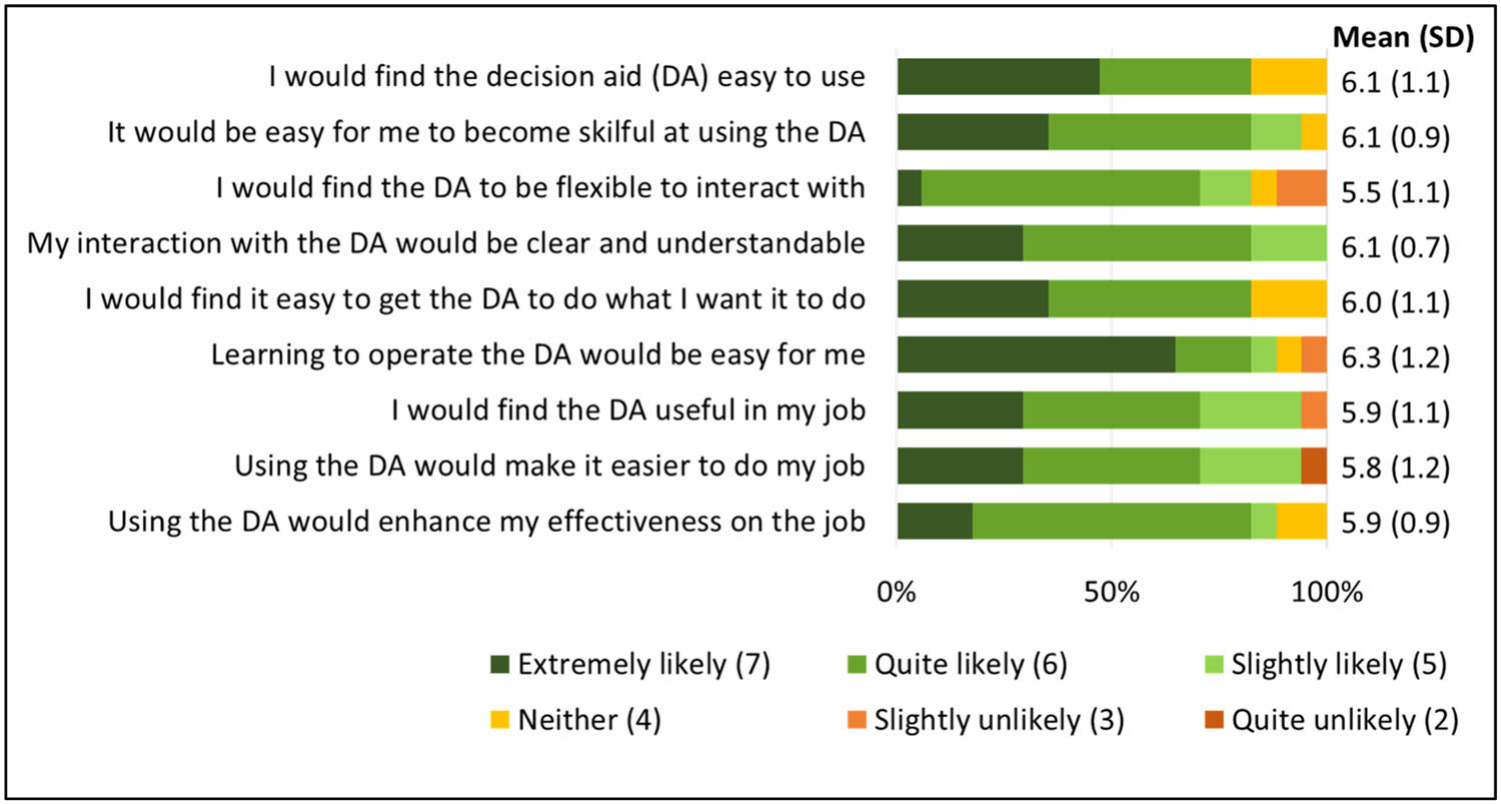
Frequency of clinician responses to usability items. Mean corresponds to a 7‐point Likert scale (1: Extremely unlikely to 7: Extremely likely). Three clinician participants did not complete the follow‐up survey.

Most clinicians felt favourably towards the decision aid even in the first round. On average, across all rounds, clinicians rated the resource 5.9 out of 7 (SD = 1.0) for perceived usefulness, and 6.0 out of 7 for perceived ease of use (SD = 0.8) (Figure 2). This positive sentiment was also reflected in the interviews:

I was thinking if I was working with a patient, how could this fit into the actual treatment—but it can definitely be easily slotted in.

(Physiotherapist, male [M], 5–9 years experience, Round 2 [R2])

Yeah, I'd use it—I think to have something like this is really helpful, especially because I find it very hard to find resources talking about surgery and interventions as an option that don't come from the people that are selling the surgery. So, I think having something that talks about it in a really even‐handed manner is really important.

(GP, female [F], 5–9 years experience, R4)

There were also opportunities to further improve clinician acceptability. For example, several clinicians felt that the ‘wait and see’ label did not adequately capture the nonsurgical management advice they typically gave. This was common feedback from chiropractors and physiotherapists who felt it would be interpreted as ‘do nothing’ rather than taking positive action to improve the pain:

I know [wait and see] is a common phrase that we use. But I guess I thought it's not really wait and see. It's kind of conservative management.

(Physiotherapist, F, 5–9 years experience, R3)

**Figure 3:**
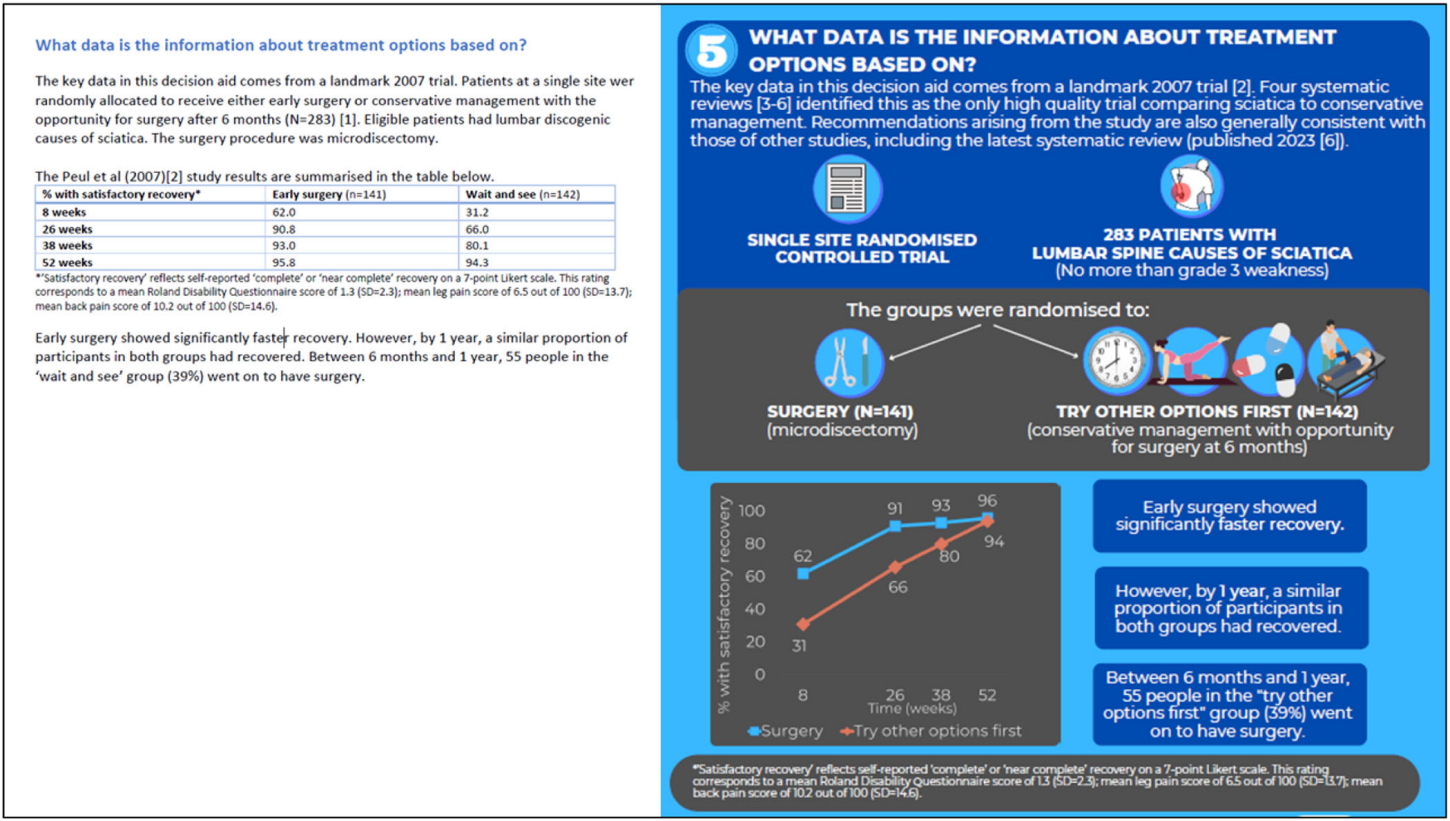
Screenshots from clinician user guide, Round 1 (left) and Round 6 (right).

To address this issue, we made iterative changes across several rounds, including adding icons to visually convey the ‘wait and see’ options in the clinician user guide, clarifying the text and ultimately, changing the label to ‘try other options first’ (Figure 3). After implementing this final change, no further concerns about the name of this option were raised.

The clinician user guide was also adapted to better support clinician acceptability. For example, after the first round, the guide was redesigned in an infographic style (Figure 3), with key messages visually reinforced. Clinicians reported they could quickly find relevant information:

The [infographics] make it easier to skim over. And I like that it's broken into the bubbles. So that's a really good way to read, it's pretty clear.

(Chiropractor, F, 2–4 years experience, R2)

Information was also added about relevant guidelines and systematic reviews to reinforce that the decision aid aligned with evidence (Figure 3).

Information about the target patients was moved to the first page of the clinician user guide to better set expectations about who the decision aid was suitable for. A few GPs and surgeons commented that the decision aid's target patient group may have limited timely access to surgery. For patients within the public health system, they reported that the target patient group may not be considered surgical candidates. For private patients, some felt the cost of surgery may be prohibitive and surgery was not a realistic option for all patients:

… in the back of my mind is always can they even access the treatment that may be recommended to them?

(GP, F, 5–9 years experience, R3)

However, even when nonsurgical management was likely to be the clinician's preferred first‐line treatment, many still saw value in showing the decision aid to patients to inform them about sciatica and outcomes for nonsurgical management.

… it's got a lot of good useful information which basically supports what I say. Saying you're probably going to get better without surgery and we only do surgery if you're desperate and you need to get better quicker because you just can't take it anymore.

(Surgeon, M, ≥ 10 years experience, R6)

#### 3.2.2 Ease of Understanding

**Figure 4:**
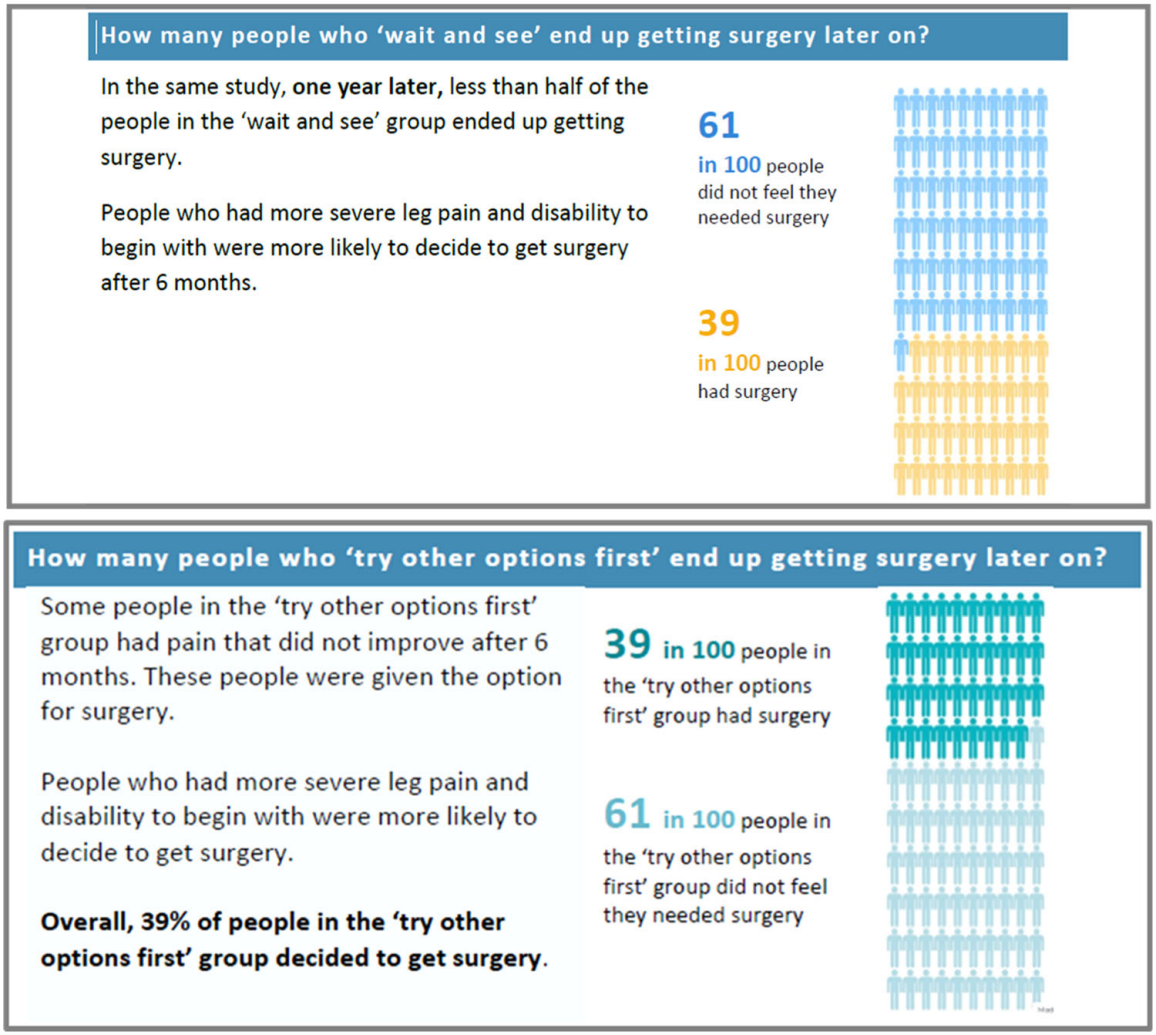
Screenshot from patient decision aid in Round 1 (top) and Round 6 (bottom).

During interviews, participants reported that most of the language in the decision aid was easy to understand, and patients appreciated the icon arrays illustrating key statistics (see Figure 4 for an example) and the glossary of common terms. The following patient explained that after a stroke several years ago, he was finding it more difficult to understand health information. For this participant, the language in the decision aid was a welcome change: it did not overwhelm and gave him confidence to make an informed decision:

I struggle with a lot of documentation because I probably [would] be panicking and sort of worried about the surgery … . the surgery will make me feel like I'm not in control, but the wording is very good … it's all fairly simple without making someone feel like they're not bright enough to understand … I'd feel more comfortable making a decision based on what I've read there.

(Patient, M, university education, 50–59 years, R7)

The validated knowledge items identified some knowledge gaps, even after reading the information (Table 3). For example, knowledge of the absolute rate of recovery for people who have surgery (Item 3) was relatively low at 50%, although most understood the gist (75% answering 70 or 90 (*n* = 15) rather than 30 or 50). Across all rounds, the average score was 3.4 out of 5 (SD = 1.2). In the final round, the average score was 3.8 (SD = 0.8, *n* = 6).

**Table 3:** Frequency for correct responses on knowledge items, *N* = 20.

Knowledge item (response options, correct answer in bold)	Number correct (%)
Item 1: Over time, without back surgery, what usually happens to back and leg pain from sciatica? (**gets better**|stays about the same|gets worse)	11 (55.0)
Item 2: Which treatment is most likely to provide faster relief from sciatica pain? (**surgery**|nonsurgical treatments|both are about the same)	14 (70.0)
Item 3: If 100 people have surgery for sciatica, about how many will have less back or leg pain 1 year after the surgery? (30|50|70|**90**)	10 (50.0)
Item 4: Sometimes surgery for sciatica does not go as planned. If 100 people have surgery for sciatica, about how many will go on to have at least one complication (e.g., needing to have surgery again)? (2|**10**|20|30)	18 (90.0)
Item 5: After several years, which treatment is better at relieving sciatic pain? (surgery|nonsurgical treatments|**both are about the same**)	14 (70.0)

Throughout the rounds of user‐testing, we identified sections of content that some patients misinterpreted and needed revision. Content was also revised if participants had to carefully read the sentence a few times to understand it:

… the bit with the blue numbers and the 61 in 100, getting me confused going, ‘Okay, so hold on, how many needed and didn't need it.

(Patient, F, university education, 40–49 years, R4, describing the section in Figure 3)

Figure 4 illustrates this issue: The text in Round 6 is less ambiguous than the text in Round 1, and links explicitly to the number in the icon array image. From Round 6, colour was also used throughout the document to help participants quickly distinguish between information about ‘try other options first’ (shown in green) and surgery (purple; not shown in Figure 4).

From Round 6, participants could more quickly and easily grasp the content conveyed in Figure 4:

To me it says that it's 39% of people … the pain was more intense for them so they changed their mind basically.

(Patient, no university education, 18–39 years, R6)

Some clinicians and patients also suggested that at 14 pages, the decision aid was too long:

Sometimes this is all really overwhelming and you need something that's really succinct to bring it back to people.

(GP, F, 5–9 years experience, R4)

In Round 5 the original text‐based summary was replaced with a one‐page infographic summary that incorporated images and colour, chunked the text and linked to other sections of the decision aid (Appendix S3, p. 6). Clinicians reported this was a more practical resource that they could print out to use during consultations and patients also appreciated having key information in one place:

… the summary is really good … because basically as a GP … I will then bring the summary … and then just present that to the patients …

(GP, F, 5–9 years experience, R4)

… I thought this [summary page] was really good the way it's got the what is, what options, how well, and what do I do next.

(Patient, F, no university education, 50–59 years, R5)

Participant feedback also indicated a strong preference for both a PDF and online (web‐based) version of the decision aid. The website version was implemented in Round 6, with some adjustment required to break up information into manageable chunks and give cues to scroll for more information. For example, one page of the decision aid depicted graphical risk information (icon arrays) for patients in both treatment groups across three time points: 2, 6 and 12 months. The graphs were shown in a 2 × 3 matrix. This worked well on an A4 PDF document as users could easily view all three rows. However, when converted to a website format, users focused on the first set of icon arrays, which showed that recovery was faster for people in the surgery group at 2 months. Often the 6‐ and 12‐month icon arrays were overlooked:

Yeah, people at first, they would think ‘Oh yes, surgery's better,’ but when you go down, it's kind of like equal, right?

(Patient, F, no university education, 18–39 years, R6)

To address this issue, key points were summarised in text at the top of the page, and users could click on boxes to see the icon arrays for each time period (2, 6 and 12 months). After making this change all remaining participants clicked through each of the boxes showing change over time.

#### 3.2.3 Patient Empowerment

**Figure 5:**
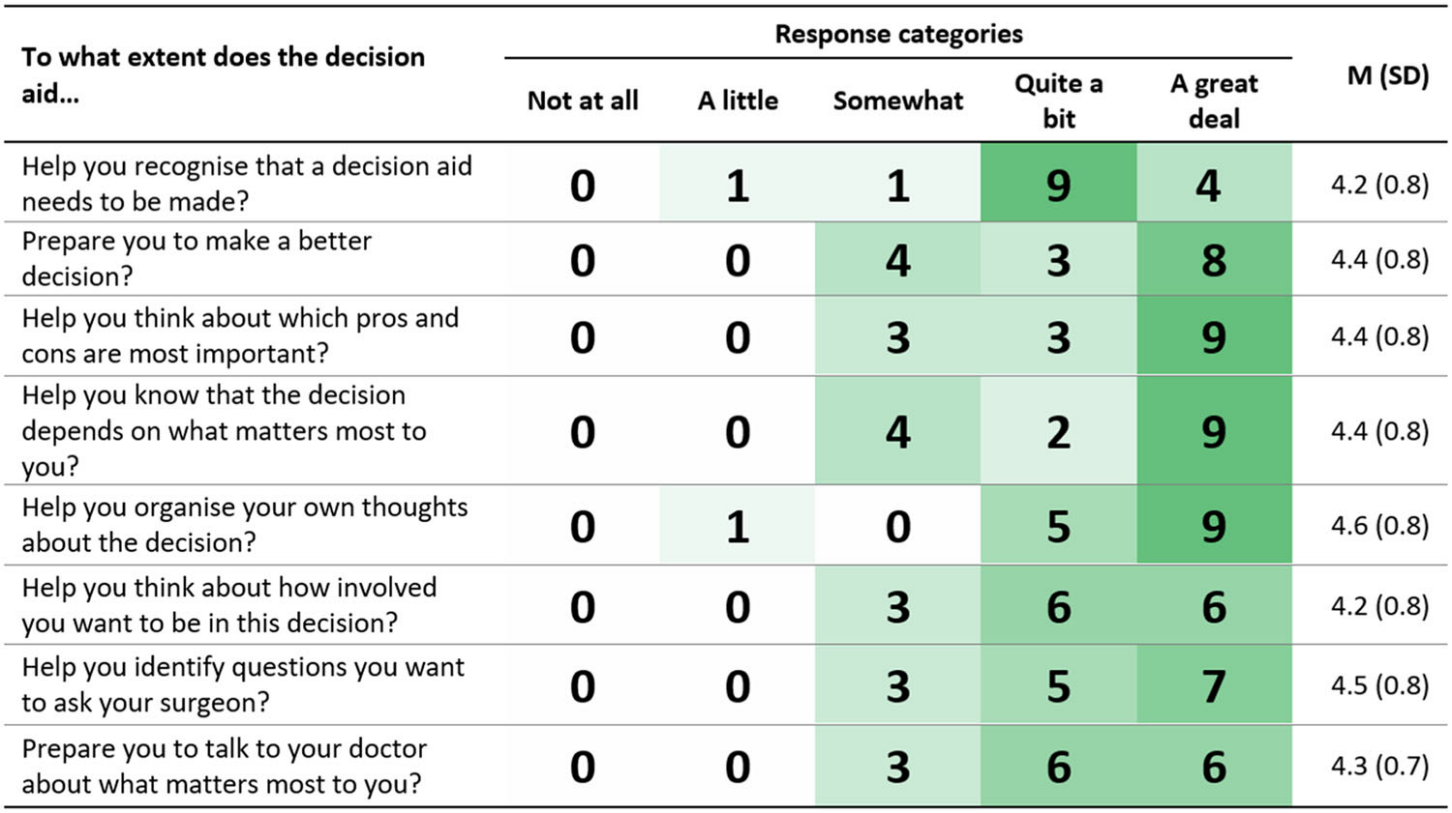
Heatmap showing frequency of patient responses to Preparation for Decision Making items, *N* = 20.

Patients and clinicians both reported that they felt the decision aid was empowering for patients. This is supported by patient responses to the Preparation for Decision Making items (Figure 5). Participants rated the items positively, on average 4.4 out of 5 (SD = 0.7). These sentiments were also evident in the interviews. Clinicians and patients discussed how the decision aid could help patients think about their values and encourage them to ask questions during medical appointments:

I think it's good the way it's got kind of—ask questions or consider your options because … it makes a person realise that there are options, there isn't only one way to go about things.

(Patient, F, no university education, 50–59 years, R5)

To enhance the question‐asking components, the text was revised to encourage readers to prioritise their most important questions, and the online version allowed users to add their own questions and download or print questions for the surgical consultation:

… they could print off … they could write, you know if they had any other further questions … [so] they're more involved in the decision‐making process

(Chiropractor, ≥10 years experience)

Text about the right to ask questions was also revised into an infographic format to emphasise the key messages:

Love this square here: ‘right to ask questions’ … You're allowed to be confident and go, ‘Help me.’ Ask them to explain—this needs to be for every medico you go and see, dentist, everything …

(Patient, F, university education, 40–49 years, R4)

**Figure 6:**
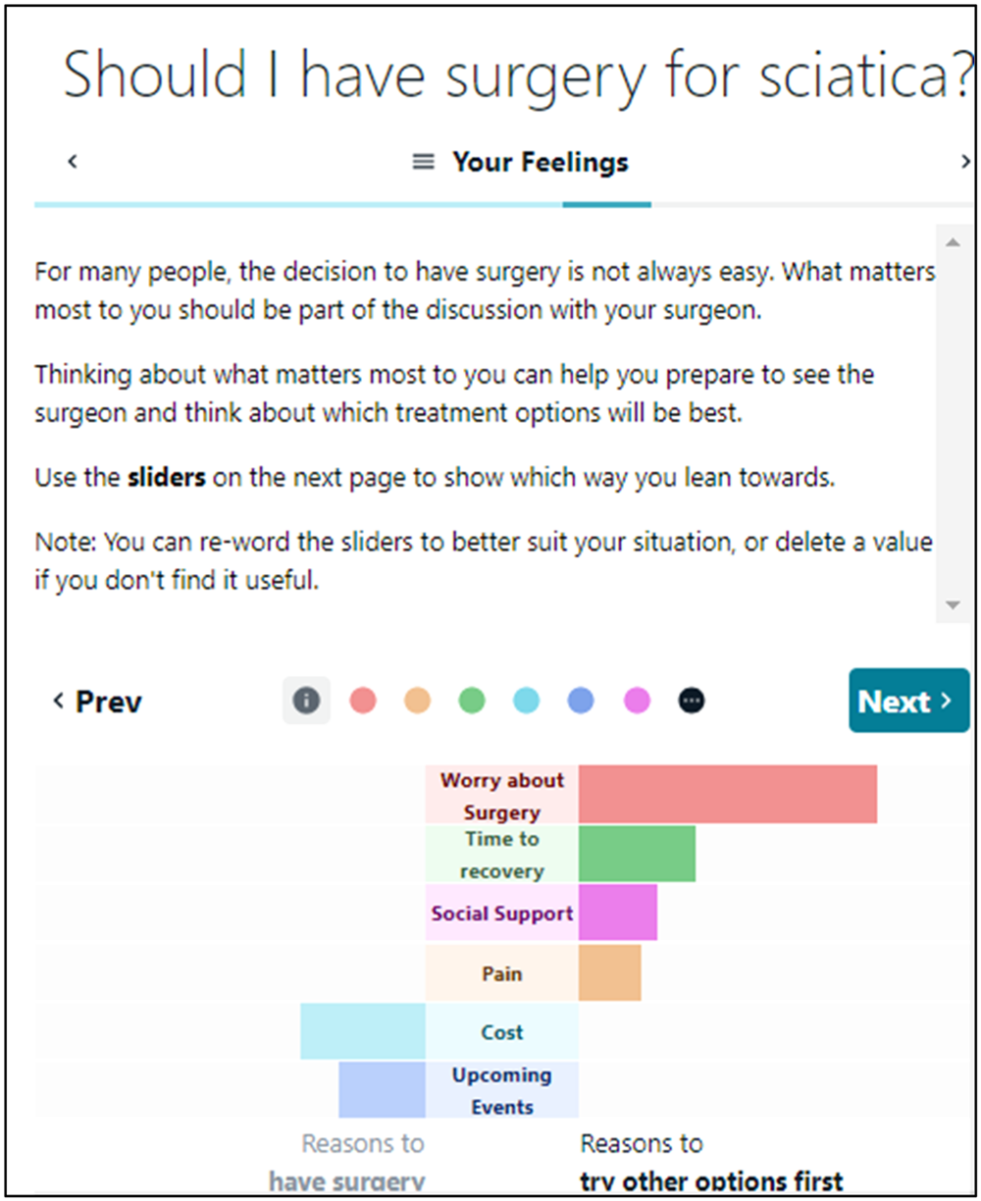
Screenshot of values clarification task instructions and completed visualisation, Round 6.

Feedback on the components that encourage reflection on personal values was overwhelmingly positive. Instructions for the values clarification task were refined over time (example shown in Figure 6). Participants were easily able to understand what to do once they moved the sliders. Most reported finding the task both interesting and useful. Participants could clearly and correctly interpret how their personalised visualisation related to their personal values and the two treatment options (surgery and try other options first):

I like it how it kind of moves the one you have the most weight into the first place [top of the visualisation], and also it kind of sums the sides up, so you see which one you're leaning towards … . So, it shows you which one is the most important to you … And also, when it moves, it's kind of fun.

(Patient, F, no university education, 18–39 years, R6)

### 3.3 Final Version

After incorporating all feedback from user‐testing, the final version of the resource comprised online and PDF (printable) versions of the patient decision aid, a clinician user guide and a printable summary sheet of key information (Appendices S3 and S4). The final version of the decision aid was written at a Grade 9.0 reading level. Though above the recommended reading level, this was due in large part to two unavoidable key words ‘sciatica’ and ‘surgery’ contributing to a higher score (Grade 7.7 with these two keywords removed). Participants in Round 7 consistently correctly interpreted the key messages during the interview and could easily engage with key website features (e.g., collapsible text, values clarification task). A link to a Word version of the decision aid was also added to the landing page to allow information to be conveyed via a screen reader.

User‐testing also provided insight into potential distribution methods. In line with participant feedback, we anticipate that time‐pressed clinicians would use the summary sheet during consultations, and patients could go through the full resource in more detail in their own time. Given its broad acceptability, the resource may be suitable for distribution by surgical clinics, GPs, physiotherapists, or chiropractors, with the aim of preparing the patient well *before* their visit to the surgeon. As emphasised in the clinician user guide, it is important that clinicians provide the resource to suitable patients, that is, those with a referral to see a surgeon.

## 4 Discussion

This study reported on the systematic development and optimisation of a new decision aid for patients considering surgery for sciatica. Codesign with key stakeholders and iterative user‐testing produced a resource that clinicians rated highly on validated measures of perceived usefulness and usability, and helped patients feel empowered and prepared to navigate the decision about whether to have surgery. Key changes identified through user‐testing related to improving clinician acceptability, ensuring content was easy to understand and empowering patients to actively engage in the decision‐making process.

This decision aid is most appropriate for a sizeable but specific subset of patients with sciatica, for whom surgical or nonsurgical management may both be appropriate treatment options. It is less suitable for patients where prompt surgery is indicated, for example, those with progressive neurological loss or cauda equina syndrome [5]. To ensure that the right patients receive the resource, we designed the decision aid so that it would be primarily delivered by a patient's health care provider. An inherent risk of this approach is poor clinician uptake, with research suggesting only 44% of decision aids continue to be used after the end of a trial [23]. However, in this study, quantitative and qualitative feedback on acceptability and feasibility has been positive, including from surgeons. We were also able to anticipate several common barriers to clinician uptake, such as poor clinician understanding of the decision aid's purpose, low feasibility in time‐poor clinical settings and ambiguity about when to introduce the decision aid to the patient [41]. Further locality‐based work is needed to develop implementation strategies that can overcome issues relating to site‐specific workflow, competing clinical priorities and buy‐in from leadership and other key stakeholders [41].

This study also highlighted the importance of incorporating health literacy into decision aid designs. In Australia, up to 60% of the population is estimated to have low health literacy [42, 43]. Despite international recognition that health literacy is important for equitable health care [44, 45, 46], it has been largely overlooked in patient decision aid research. A systematic review of 213 trials of patient decision aids reported that only 25 decision aids (11.7%) considered the needs of people with low health literacy or people from socially disadvantaged communities [26]. In this study, we sought to address this issue by using health literacy strategies such as plain language, white space and shortening long sections of text. Applying these kinds of health literacy strategies has been shown to improve knowledge, comprehension and perceptions of risk [30, 31, 47, 48]. Our participants also reported additional benefits such as feeling less overwhelmed and more confident about engaging in health decisions.

In addition to presenting information about treatment options, decision aids should also aim to guide patients through the decision‐making process [32]. This is particularly important for this decision aid given the documented power imbalance between surgeons and patients with sciatica that can reduce patient confidence to ask questions and discuss their personal preferences [13, 49]. Evidence suggests the question‐asking components in this decision aid (e.g., question prompt list) can help patients take a more active role in health decision‐making and improve communication processes [50, 51, 52, 53], and user‐testing helped refine these further. To help patients reflect on their values, the decision aid included an interactive task. Rating scales are a common feature in decision aids and are an effective way to reduce conflicted feelings about a health decision [28, 54]. However, experimental evidence suggests that rating scales may be further enhanced by explicitly showing patients how the ratings align with different treatment options; this is also a format that patients prefer [27]. In this study, we developed a promising prototype that received consistently positive feedback, and that we hope can further advance this field of research.

This study had several strengths. First, the decision aid benefited from a working group and user‐testing study that helped integrate end‐user perspectives into its design, and both of these components included clinicians and patients with varied clinical and sociodemographic experiences. Codesign and rigorous user‐testing are increasingly recognised as essential for developing digital health interventions that are usable, engaging and more likely to be used in real‐world settings [55, 56, 57]. Further research could evaluate how the decision aid impacts patient outcomes.

A key limitation is that some adaptations may have been useful but were beyond the scope of this project. For example, all participants tested the decision aid on a computer screen. Further testing and adaptation may be needed to ensure that it is mobile friendly. Further adaptation may also be needed to meet the needs of different priority groups such as people with disabilities and people from culturally and linguistically diverse backgrounds. It is unknown how people with current severe sciatica would engage with the decision aid.

## 5 Conclusion

Many patients with sciatica may consider the option to have surgery. We developed a decision aid to help patients navigate this decision with their surgeon. Codesign and user‐testing were crucial for ensuring that the decision aid met end‐user needs. This study demonstrated that the decision aid was acceptable to a variety of clinicians (surgeons, GPs, physiotherapists, and chiropractors), can equip patients with the information they need in a format they can understand and can give patients confidence to take a more active role in decision‐making.

## Author Contributions

**Julie Ayre:** conceptualisation, investigation, writing–original draft, methodology, writing–review and editing, formal analysis, project administration, resources, data curation. **Richie Kumarage:** investigation, formal analysis, project administration, writing–review and editing. **Hazel Jenkins:** conceptualisation, writing–review and editing, methodology. **Kirsten J. McCaffery:** writing–review and editing, conceptualisation, supervision, methodology. **Christopher G. Maher:** conceptualisation, writing–review and editing, funding acquisition, methodology. **Mark J. Hancock**: conceptualization, funding acquisition, writing–review and editing, supervision, methodology.

## Ethics Statement

Ethical approval for the study was obtained from the University of Sydney Human Research Ethics Committee (Project number 2022/678).

## Consent

Participants reviewed the participant information sheet and completed online consent forms before taking part in the study.

## Conflicts of Interest

Julie Ayre and Kirsten J. McCaffery are directors of a health literacy consultancy (Health Literacy Solutions Ltd, Pty). The company provides health literacy advice to health services/organisations to support increased access to health information for low literacy adults. Any revenue raised is used to support the development of tools to support health literacy document design. No personal income is received by Julie Ayre or Kirsten J. McCaffery.

## Supporting information

Appendix 1: Patient recruitment flyer.

Appendix 2: Patient screening survey.

Appendix 3: Patient decision aid (PDF version).

Appendix 4: Clinician user guide.

Appendix 5: Participant data.

## Data Availability

The data that support the findings of this study are available in the Supporting information of this article.
