# Supplementary material for: A Decision Aid for Patients Considering Surgery for Sciatica: Codesign and User‐Testing With Patients and Clinicians
Source: Health Expect. 2024 Jun 19;27(3):e14111. doi: 10.1111/hex.14111 (PMC11186058; doi:10.1111/hex.14111)
Supplement: Supplementary file 1 — Appendix 1: Patient recruitment flyer. [file HEX-27-e14111-s002.pdf]

THE UNIVERSITY OF
SYDNEY

Interview study

We want to hear from people with **sciatica** or **low back pain**

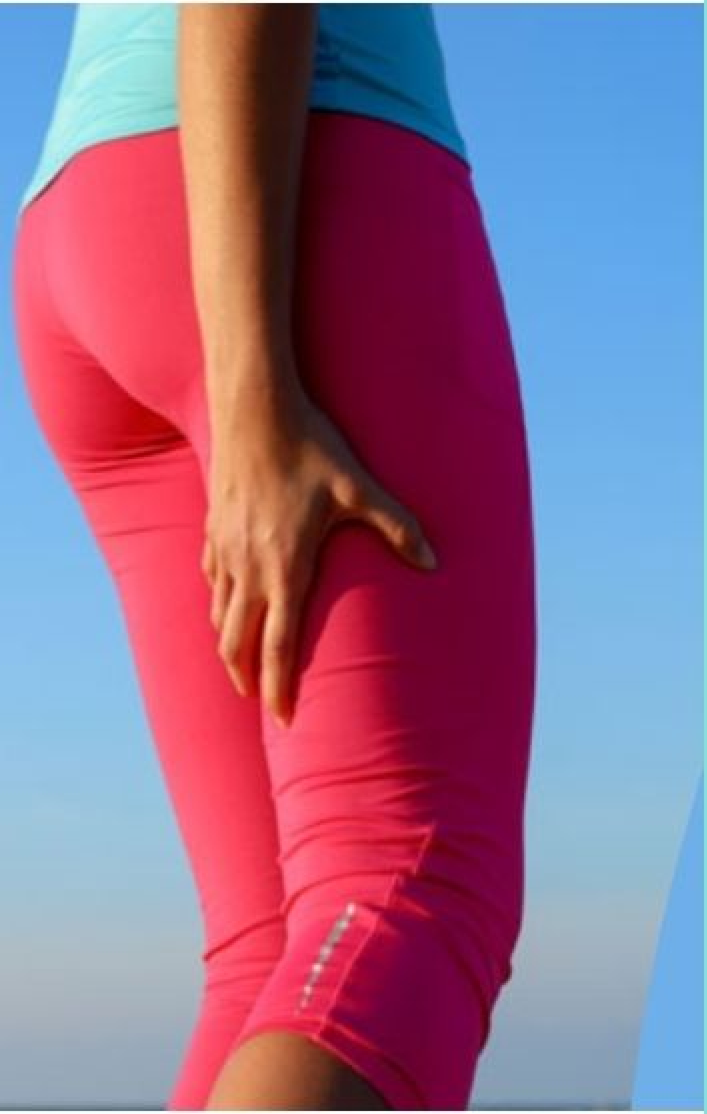

Researchers from the University of Sydney are looking for people with **sciatica** or **low back pain** (past or present) to take part in an online interview via Zoom.

We will ask people to test out a new **interactive decision tool** that helps people:

- **learn** about sciatica and different treatment options
- **think** about what matters most
- **decide** which option suits them best

Interested? Click the survey link to find out more and register.

ANZBACK
AUSTRALIA & NEW ZEALAND
LOW BACK PAIN RESEARCH NETWORK
NHMRC CENTRE OF RESEARCH EXCELLENCE
