# Supplementary material for: A Decision Aid for Patients Considering Surgery for Sciatica: Codesign and User‐Testing With Patients and Clinicians
Source: Health Expect. 2024 Jun 19;27(3):e14111. doi: 10.1111/hex.14111 (PMC11186058; doi:10.1111/hex.14111)
Supplement: Supplementary file 2 — Appendix 2: Patient screening survey. [file HEX-27-e14111-s005.docx]

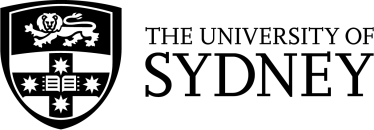

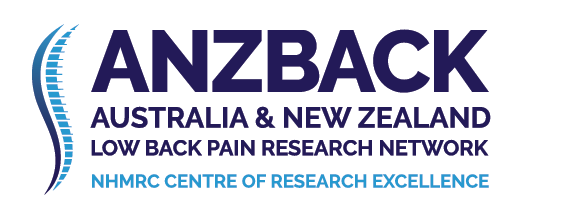


**Interactive decision aid for patients considering surgery for sciatica:**

**user-testing study**

**Screening survey for patients**

**What is this study about?**

Researchers at the [removed] are testing a new online interactive tool that helps people learn about sciatica and their treatment options. The tool also helps people with sciatica think about whether surgery is right for them. It does this by helping people reflect on what matters most. The findings from this study will help us make sure the tool is easy to understand and use.

**What will I be asked to do?**

This study has three parts:

- **Survey 1:** A 5-minute survey that will ask if you would like to take part, your contact details, and a few questions about yourself like age, gender, your sciatica.
- **Interview**: We will ask you to try out the tool and ask some questions afterwards
- **Survey 2:** A 5-minute survey that will ask how you felt about the tool

We will use the information from survey 1 to make sure we interview people with a range of different experiences. This means that not everyone who completes the survey will take part in an interview.

If you are invited to an interview, we will ask you to use the tool and ‘think aloud’ as you go. We will also ask you about:

- How this tool might help you make a decision about surgery for sciatica
- What you think of the tool
- How we could improve it

The interview will take about 45 minutes and you can do this at a time that suits you. We will use Zoom to record the interview.

You can take part if you are:

- over 18, live in Australia, and can speak, read and understand English
- have sciatica/low back pain or had it in the past

As a token of appreciation for your contribution, those who are interviewed will also receive a $40 gift voucher.

Being in this study is completely voluntary and you do not have to take part. Your decision whether to participate will not affect your current or future relationship with the researchers, anyone else at the University of Sydney or anyone who is associated ANZBACK. It will not affect your relationship with your treating clinician, and they will not know whether you decide to take part in the study.

To read more information about this part of the study, please download the Participant Information Statement <link to PDF version of PIS>.

I confirm that I have read the Participant Information Statement and consent to take part in this research project as described.

| Yes | No |
| --- | --- |

I understand that if invited to take part in an interview, my participation may be audio and/or video-recorded.

| Yes | No |
| --- | --- |

[If ‘yes,’ to both of the above questions, proceed to survey question on next page, if ‘no,’ survey ends and no data is collected.]

# [Screening items]

How old are you?

____________

Which state do you live in?

| Australian Capital Territory |
| --- |
| Northern Territory |
| New South Wales |
| Victoria |
| Queensland |
| Western Australia |
| South Australia |
| Tasmania |
| I do not live in Australia |

Which best describes your pain now?

- Back pain only
- Back pain and related leg pain that extends into the thigh, but does not pass the knee
- Back pain and related leg pain that extends below the knee
- None of the above

Which best describes your pain in the past?

- Back pain only
- Back pain and related leg pain that extends into the thigh, but does not pass the knee
- Back pain and related leg pain that extends below the knee
- None of the above

How much did your **worst episode of back pain** interfere with your normal activities daily activities?

- Not at all
- A little bit
- Moderately
- Quite a bit
- Extremely

[Screening criteria: agree to consent, live in Australia, >18 years, some experience of low back pain/sciatica, worst episode had at least moderate level of interference with normal daily activities.

If participants do not meet screening criteria they will not continue with the survey]

# [survey items]

What is your gender?

| Male | Female | Other (please tell us) | Prefer not to say |
| --- | --- | --- | --- |

What is your highest level of education?

| Less than high school |
| --- |
| High school graduate |
| Certificate, or equivalent |
| Diploma, bachelor degree or equivalent |
| Masters or Doctoral degree, or equivalent |

In which country were you born?

| Australia | Other (specify) |
| --- | --- |

[If not Australia]: In what year did you move to Australia to live? __________________

Are you of Aboriginal and/or Torres Strait Islander origin? No Yes Prefer not to say

How confident are you filling out medical forms by yourself?

| Extremely |
| --- |
| Quite a bit |
| Somewhat |
| A little bit |
| Not at all |

Do you have young children (<18 years)?

| Yes | No |
| --- | --- |

[If yes] Please list how many and their ages (do not include names):

___________________________________________________

Has your doctor ever told you that you have?

| Rheumatoid arthritis | Depression | Asthma |
| --- | --- | --- |
| Arthritis | Anxiety | Diabetes |
| Osteoarthritis | Stroke | COPD (lung disease) |
| Other muscle or joint pain | Heart disease | Cancer |
| Headache or migraine | Kidney disease | Other (specify) |

Thank you for taking part in this study! Would you like to receive a summary of study findings:

| Yes | No |
| --- | --- |

Please provide an email address or phone number so we can contact you to arrange the interview. To receive a summary of study findings you will need to provide an email address.

Email: _____________________________________

Phone: ____________________________________
