# Supplementary material for: A Decision Aid for Patients Considering Surgery for Sciatica: Codesign and User‐Testing With Patients and Clinicians
Source: Health Expect. 2024 Jun 19;27(3):e14111. doi: 10.1111/hex.14111 (PMC11186058; doi:10.1111/hex.14111)
Supplement: Supplementary file 3 — Appendix 3: Patient decision aid (PDF version). [file HEX-27-e14111-s003.pdf]

Should I have surgery for sciatica?

How can this website help you?

This website is for you if:

✓ Your health professional has referred you to a surgeon for an opinion

AND

✓ You have had leg pain from sciatica

This website will help you make the most out of seeing a surgeon. There are three parts:

Before you visit the surgeon

- Learn about sciatica
- Learn about your options
- Think about what you need to ask the surgeon
- Think about what matters most to you

During the visit to the surgeon

- The surgeon will tell you whether surgery is likely to help your sciatica
- Talk to the surgeon about what matters most to you
- Ask questions to make sure you understand your options.

After you visit the surgeon

- Consider your options
- Think about the evidence, what matters most to you, and the surgeon's advice
- Make a decision about surgery

Read through the website and then talk to your surgeon about your options. Once you know your options you can think about what matters most to you. You can also talk to a health professional to help find the right choice for you. This might be your GP, your physiotherapist, your chiropractor or the surgeon. You can show them the website and ask more questions.

Scan QR code to view online!

ANZBACK AUSTRALIA & NEW ZEALAND LOW BACK PAIN RESEARCH NETWORK NHMRC CENTRE OF RESEARCH EXCELLENCE

THE UNIVERSITY OF SYDNEY

MACQUARIE University

Before you visit the surgeon

- Learn about sciatica
- Learn about your options
- Think about what you need to ask the surgeon
- Think about what matters most to you

What is sciatica?

Sciatica is pain that extends from the lower back, into the leg, usually below the knee and sometimes into the foot and toes. This is also commonly called radicular pain. For most people, this pain happens when a disc in the spine is irritating one of the nerves that runs from the spine into the leg. This pain is different from person to person.

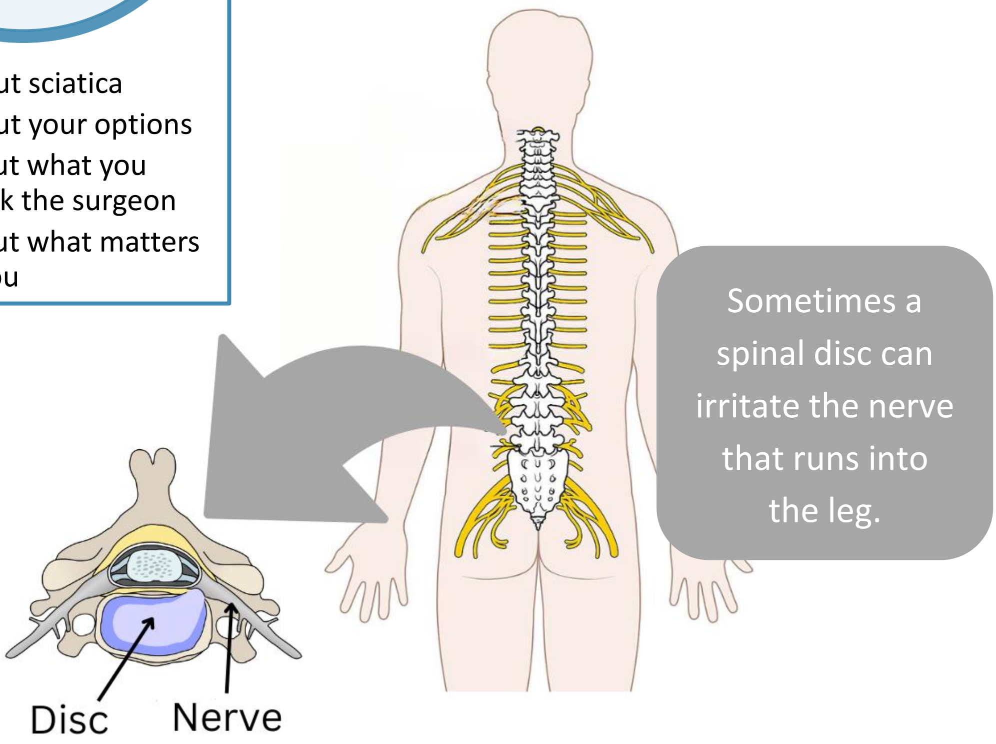

What does sciatica feel like?

The pain from sciatica can range from very mild to very strong. Often people feel a burning, tingling or sharp feeling, while for others it is an ache. Some people also feel numbness, pins and needles or weakness in the leg or foot.

How does sciatica change over time?

Most people with sciatica start to improve within six weeks without any intervention. Most people's pain does not get worse. After three months:

- Most have much less pain
- Most no longer use medicine to manage the pain
- Most are able to return to leisure activities (e.g. hobbies or sport)

What options might the surgeon suggest?

If your pain is not improving quickly on its own, your surgeon may suggest two main options.

Surgery 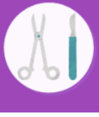	Try other options first 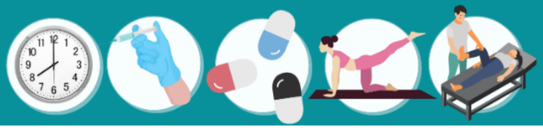
**Have surgery**	**Try other options first and see if your pain improves. If the pain doesn't improve, you can still decide to get surgery.**
Having surgery for sciatica means<br>• Going to hospital (usually for 2 – 3 days)<br>• Rehabilitation (e.g. physical therapy, exercise)	There are different options for managing your sciatica. Ask your clinician about:<br>• Pain medicines<br>• Exercises<br>• Manual therapy of the spine<br>• Acupuncture<br>• Injections
**Reasons to choose this option**<br>• Your pain may improve more quickly<br>• You may return more quickly to doing thing that matter to you (e.g. work or hobbies)	**Reasons to choose this option**<br>• You may get better on your own. You may not need surgery.<br>• You can take more time to make a decision about surgery<br>• You may find other benefits to physical therapy<br>• You may avoid the cost of surgery
**Reasons to avoid this option**<br>• Cost of surgery and other care or medicine<br>• Complications from surgery (e.g. bleeding), risk from anaesthesia<br>• Does not guarantee full recovery. The pain may return and you may need surgery again	**Reasons to avoid this option**<br>• Your pain may improve more slowly<br>• Cost of treatment or medicine<br>• You may need surgery later<br>• You may have side effects from the medicine. How likely you are to have side effects will depend on the medicine you use

How well does surgery for sciatica work?

There is one high quality study that looks at how well surgery for sciatica works. This study compared **people who had surgery** and **people who tried other options first.** The main findings were that:

- In the **first few months**, pain improved more quickly for people who had surgery.
- By **12 months**, most people in both groups were free of pain (about 95% of people).

**Surgery***	**Try other options first†**

2 months later

62 **in 100** people who were offered **surgery** were free of pain*

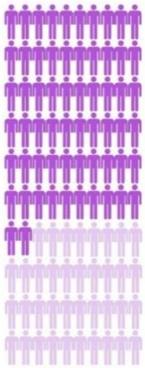

31 **in 100** people who were asked to **try other options first** were free of pain†

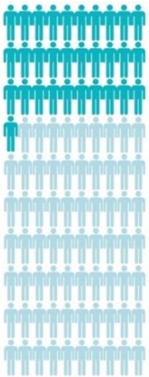

6 months later

91 **in 100** people who were offered **surgery** were free of pain*

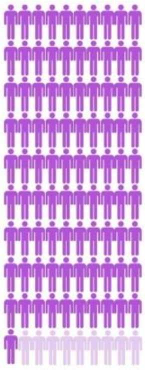

66 **in 100** people who were asked to **try other options first** were free of pain†

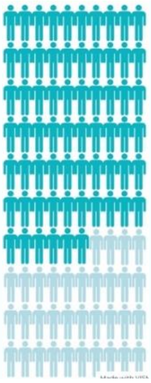

1 year later

96 **in 100** people who were offered **surgery** were free of pain*

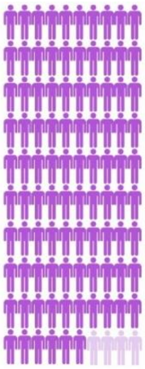

94 **in 100** people who were asked to **try other options first** were free of pain†

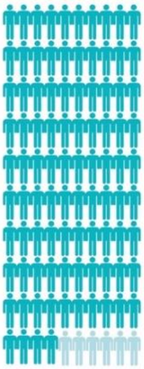

*Counts for the 'surgery' group include 11 people who recovered from sciatica before they could have the surgery

† The people in the 'try other options first' group tried other options such as pain medicines, exercise, and manual therapy of the spine.

How many people who 'try other options first' end up getting surgery later on?

Some people in the 'try other options first' group had pain that did not improve after 6 months. These people were given the option for surgery.

People who had more severe leg pain and disability to begin with were more likely to decide to get surgery.

Overall, 39% of people in the 'try other options first' group decided to get surgery.

6 months later

39 in 100 people in the 'try other options first' group had surgery

61 in 100 people in the 'try other options first' group did not feel they needed surgery

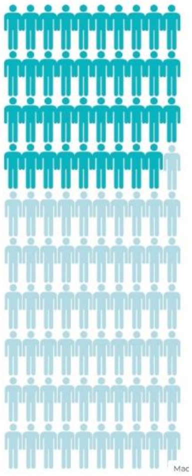

What are the risks of surgery?

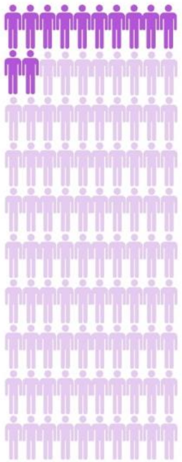

12 in 100 people will have serious complications from surgery

88 in 100 people will not have serious complications from surgery

About **88%** of people who have surgery for sciatica do not go on to have any serious complications.

Sometimes though, the surgery does not go as planned. About **12%** of people who have surgery for sciatica go on to have at least one serious complication.

For example, some people:

- May need to have surgery again (3.7% to 10.2%). How likely this is will depend on the type of surgery.
- May have less movement or sensation, or new sciatica pain (3.0%)
- May have complications such as heart attack, problems with lungs or kidneys, and difficulty breathing (2.6%)
- May have damage to the spinal tissue (4.5%)

The numbers above do not include other, less serious issues that can happen when you have surgery. These other issues are also generally more common. Examples are nausea and vomiting, cough, and constipation.

What happens in the long term for people who have surgery?

In the section above, we showed that most people are free of pain one year after they have surgery.

- For the next 2-3 years after the surgery, the pain may become a bit worse. But usually the pain is not as bad as the person had before the surgery.

- There is a similar pattern for people who try other options first. Most people recover in the first year. The pain may become a bit worse after that, but is usually not as bad as when the pain first started.

SHOULD I HAVE SURGERY FOR SCIATICA?

SUMMARY OF KEY INFORMATION

1 WHAT IS SCIATICA?

- Sciatica is pain that extends from the lower back, into the leg, usually below the knee and sometimes into the foot and toes. For most people, this pain happens when a disc in the spine is irritating one of the nerves that runs from the spine into the leg
- Often people feel a burning, tingling or sharp feeling, while for others it is an ache. Some people also feel numbness, pins and needles or weakness in the leg or foot.
- Most people with sciatica start to improve within six weeks without any intervention.

Read more page 2

2 WHAT OPTIONS MIGHT THE SURGEON SUGGEST?

1) Surgery

Going to hospital for surgery, along with rehabilitation exercises.

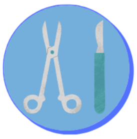

Read more page 3

2) Try other options first:

You can try other options first, and see if your pain improves over time. Ask your clinician about your options:

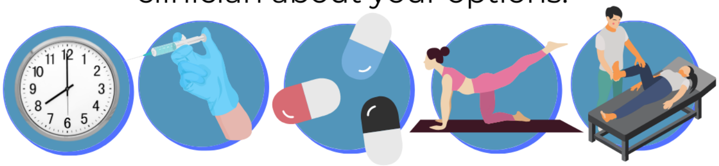

If the pain doesn't improve, you can still decide to get surgery later on.

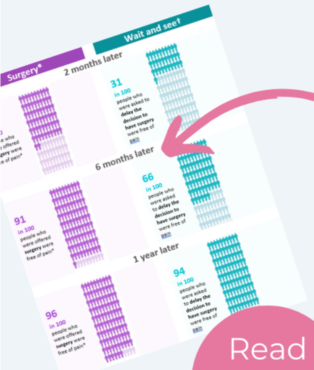

Read more page 4

3 HOW WELL DOES SURGERY FOR SCIATICA WORK COMPARED TO TRYING OTHER OPTIONS FIRST?

- Having surgery can help your leg pain improve faster in the first few months.
- By 12 months, most people in both groups (surgery and try other options first) are free of pain (about 95%).
- About 12% of people who have surgery for sciatica go on to have serious complications.
- After one year, most people who try other options first do not end up needing surgery (61%).
- Having surgery does not guarantee that the pain will go away. A small number of people need surgery more than once (up to 10%).
- In the long term, both treatment options are about the same.

4 WHAT DO I DO NEXT?

See the full resource using the QR code

Go to page 7 to help you think about what matters most.
Go to page 8 to read patient stories.

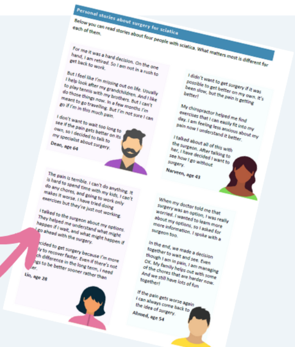

Read more page 8

What matters most to you?

For many people, the decision to have surgery is not always easy. What matters most to you should be part of the discussion with your surgeon. Thinking about what matters most to you can help you prepare to see the surgeon and think about which treatment options will be best.

Below are some things you could think about before you see your surgeon:

Reasons to have surgery	Reasons to try other options first
If there's a chance that I have to have surgery I'd rather have it sooner than later.	I would rather avoid having surgery if I can get better on my own.
I am in a lot of pain. I don't see how I can stand it much longer.	My pain isn't bad enough that I need to have surgery right now.
It's very important that I get my pain under control so that I can go back to things that matter as soon as possible (e.g. work, family, hobbies).	Time is not a problem for me. If I get better slowly using exercises and/or medicine, that's okay with me.
I'm not worried about how much this surgery will cost.	I want to avoid unnecessary medical costs if I can.
There are some big events or activities in the next few months that I will find difficult with this pain	There are no big events or activities in the next few months that I will find difficult with this pain
I will have trouble finding people who can help out with everyday tasks	I have people around me who can help with everyday tasks e.g. shopping, travel

You may also have other important reasons to have surgery or try other options first.

On the next page you can read stories about four people with sciatica. What matters most is different for each of them.

The next step is to see the surgeon. They will talk to you about whether surgery is likely to help your sciatica. Together you can decide if surgery is right for you.

Personal stories about surgery for sciatica

Below you can read stories about four people with sciatica. What matters most is different for each of them.

For me it was a hard decision. On the one hand, I am retired. So I am not in a rush to get back to work.

But I feel like I'm missing out on life. Usually I help look after my grandchildren. And I like to play tennis with my brothers. But I can't do those things now. In a few months I'm meant to go travelling. But I'm not sure I can go if I'm in this much pain.

I don't want to wait too long to see if the pain gets better on its own, so I decided to talk to my specialist about surgery.

Dean, age 64

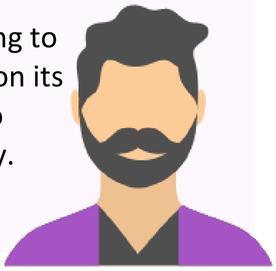

I didn't want to get surgery if it was possible to get better on my own. It's been slow, but the pain is getting better!

My chiropractor helped me find exercises that I can easily fit into my day. I am feeling less anxious about my pain now I understand it better.

I talked about all of this with the surgeon. After talking to her, I have decided I want to see how I go without surgery.

Narveen, age 43

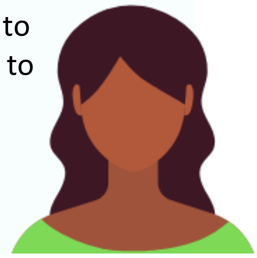

The pain is terrible. I can't do anything. It is hard to spend time with my kids, I can't do any chores, and going to work only makes it worse. I have tried doing exercises but they're just not working.

I talked to the surgeon about my options. They helped me understand what might happen if I wait, and what might happen if I go ahead with the surgery.

I decided to get surgery because I'm more likely to recover faster. Even if there's not much difference in the long term, I need things to be better sooner rather than later.

Lin, age 28

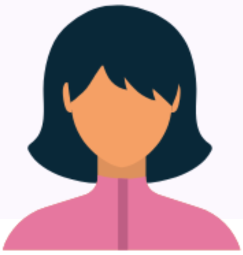

When my doctor told me that surgery was an option, I was really worried. I wanted to learn more about my options, so I asked for more information. I spoke with a surgeon too.

In the end, we made a decision together to wait and see. Even though I am in pain, I am managing OK. My family helps out with some of the chores that are harder now. And we still have lots of fun together!

If the pain gets worse again I can always come back to the idea of surgery.

Ahmed, age 54

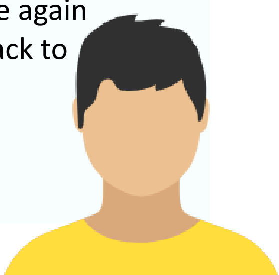

During the visit to the surgeon

- The surgeon will tell you whether surgery is likely to help your sciatica
- Talk to the surgeon about what matters most to you
- Ask questions to make sure you understand your options

What happens when I visit the surgeon?

When you see the surgeon, they will tell you whether surgery is likely to help your sciatica.

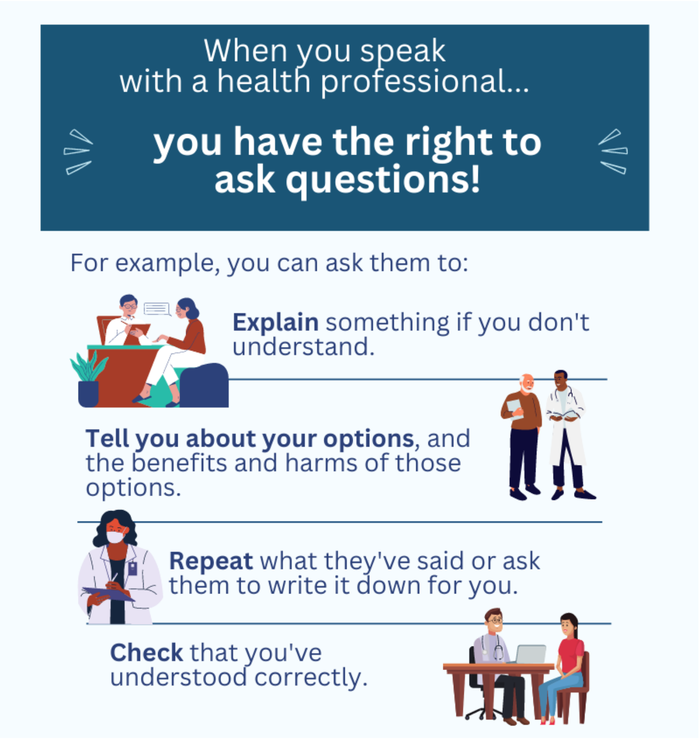

You can also talk to them about what matters most to you.

When you go to see a surgeon you may like to ask:

- ☐ What are the other options? (cortisone injection, physical therapy, exercises, medications etc.)
- ☐ If I decide to try other options first, what should I expect with my pain levels?
- ☐ There are a few studies that have looked at whether surgery for sciatica works. How well does that research apply to me and my sciatica? (You can show them this website!)
- ☐ Can you tell me more about what happens during surgery?
- ☐ Can you tell me more about the risks that come with the surgery? How likely are these risks?
- ☐ How long will I have to wait for the surgery?
- ☐ Is there anything I need to do before or after surgery, such as physical therapy?
- ☐ If I have surgery, when would I be able to go back to work?

Add your own questions below:

....

....

Tip: To get the most out of your visit with the surgeon, think about **which questions are most important to you.**

After you visit the surgeon

- Consider your options
- Think about the evidence, what matters most to you, and the surgeon's advice
- Make a decision about surgery

I've talked to the surgeon. What are my next steps?

If your surgeon suggests that surgery is an option for you, you can decide whether to have surgery or try other options first, to see if your pain improves by itself.

For many people, the decision to have surgery will not always be clear. After seeing the surgeon, you can reflect on the information from this website, the information the surgeon provided and what matters most to you.

Use our **interactive tool** to help you make your decision:

Scan QR code to view!

What have you decided? Remember, there is no right or wrong answer.

- ☐ I would like to have surgery
- ☐ I am unsure
- ☐ I would like to try other options first

It's also ok if you want to get a **second opinion** from another specialist. For some people, this helps them decide. You can ask your GP about who to contact.

Do I have to get surgery?

No, having surgery is your decision.

I am unsure about what to do

If you're unsure about what to do, you may wish to read through the website again or talk to someone about your remaining questions. You can talk about the decision with family and friends, or your doctor, physiotherapist or chiropractor. Remember, if the pain doesn't improve, you can still decide to get surgery later.

I have made a decision

If you decided to try other options first, you can talk to your doctor, physiotherapist or chiropractor about how to manage the pain. If you decided to get surgery, talk to your surgeon for next steps.

Where can I learn more?

Websites to learn more about sciatica

1. **Australian website:** https://www.healthdirect.gov.au/sciatica
2. **US website:** https://my.clevelandclinic.org/health/diseases/12792-sciatica
3. **UK website:** https://www.nhs.uk/conditions/sciatica/

Research studies used in this website

Below are some of the main studies that this website uses.

How does sciatica change over time without treatment? (see page 2)

Weber H, Holme I, Amlie E. The natural course of acute sciatica with nerve root symptoms in a double-blind placebo-controlled trial evaluating the effect of piroxicam. Spine (Phila Pa 1976). 1993;18(11):1433-8. Epub 1993/09/01. PubMed PMID: 8235813. https://pubmed.ncbi.nlm.nih.gov/8235813/

Summary: In this study, 208 patients with sciatica either had a medicine called piroxicam or placebo (medicine with no active ingredient). Piroxicam had the same effects as placebo, so we can use the study to look at how sciatica changes over time without treatment. By 3 months, 76% of patients reported 'satisfactory back function.' After 12 months this was 70%.

How well does surgery for sciatica work, for people who've had sciatica for less than 4 months? (page 4)

Peul WC, van Houwelingen HC, van den Hout WB, Brand R, Eekhof JAH, Tans JTJ, et al. Surgery versus Prolonged Conservative Treatment for Sciatica. New England Journal of Medicine. 2007;356(22):2245-56. https://pubmed.ncbi.nlm.nih.gov/17538084/

Summary: Researchers randomised 283 patients to have surgery (141 patients) or 'try other options first' (142 patients). Patients in the 'try other options first' group tried options such as pain medicines, exercise, and manual therapy of the spine. After 6 months they could get surgery if they wanted. You can read about the study findings in the sections '*How well does surgery for sciatica work?*' and '*How many people who try other options first end up getting surgery later on?*'

How common are complications from surgery for sciatica? (page 5)

Shriver MF, Xie JJ, Tye EY, Rosenbaum BP, Kshettry VR, Benzel EC, et al. Lumbar microdiscectomy complication rates: a systematic review and meta-analysis. Neurosurgical Focus FOC. 2015;39(4):E6. doi: 10.3171/2015.7.FOCUS15281. https://pubmed.ncbi.nlm.nih.gov/26424346/

Summary: This review looks at 42 articles about surgery for sciatica. It reports on how often complications happen. You can read more in the 'section '*What are the risks of surgery?*'

How well does surgery for sciatica work in the long term? (page 6)
Machado GC, Witzleb AJ, Fritsch C, Maher CG, Ferreira PH, Ferreira ML. Patients with sciatica still experience pain and disability 5 years after surgery: A systematic review with meta-analysis of cohort studies. European Journal of Pain. 2016;20(10):1700-9. https://pubmed.ncbi.nlm.nih.gov/27172245/

Summary: This review looks at 40 studies to understand how pain and disability changes over time, for patients who have had surgery for sciatica. You can read more about the study findings in the section '*What happens in the short and long term for people who have surgery?*'

How was this tool developed?

This decision aid was developed by Dr Julie Ayre (BLibSt (Hons), PhD), Prof Mark Hancock (BAppSci(Phty), MAppSc, PhD), Dr Hazel Jenkins (MChir, MAppSci (Medical Imaging), BMedSci, PhD), Prof Christopher Maher (BAppSc (Phty), PhD, DMedSc, FACP, FAAHMS), and Prof Kirsten McCaffery (BSc(Hons), PhD).

Development of this tool was supported by the Australia and New Zealand Low Back Pain Research Network (ANZBACK) a Centre of Research Excellence funded by the National Health and Medical Research Council (APP1171459). No conflicts of interest declared.

We would like to acknowledge the contributions of our stakeholder working group, comprising Dr Ben Ewald, Dr Gemma Abraham, Dr Ahmad Hosseini, Ms Narveen Kaur, Mr Ben Cronin, Mr Chris Agius, Prof Kevin Seex, Prof Randolph Gray, Prof Gavin Davis, Prof Susan Liew, Prof Kate Drummond, Ms Louise Gross, Mr Ajay Varshney, Mr Steven Durrell, Mr Ang Montesano, and Ms Karen Tambree. We would also like to acknowledge the support of Prof Ian Harris and Ms Deborah Fullwood (ANZBACK).

Publication date: 19 September 2023.

Sciatica and spine images:

Debivort at en.wikipedia, CC BY-SA 3.0, via Wikimedia Commons

Andrewmeyerson, CC BY-SA 4.0, via Wikimedia Commons

Terms your clinician might use

Annulus fibrosis – the harder outer part of the disc

Cauda Equina syndrome – a serious condition that occurs when there is compression of the nerves at the lower end of the spinal cord and can affect bowel and bladder function. This is a rare condition but if found requires immediate attention.

Claudication pain – leg pain that is worse when walking/being active and results from a lack of blood supply through the arteries. This is sometimes confused with sciatica.

Dermatomes – areas of the skin (sensation) supplied by a specific nerve coming from the spine

Disc degeneration: A process where the discs in the spine become less hydrated and lower in height. This is often a normal part of aging.

Disc herniation – occurs when the nucleus (softer centre part of the disc) pushes through the annulus (harder outer part of the disc). Terms like protrusion, extrusion, and sequestration describe different types of herniations.

Erector spinae muscles – the main group of muscles that surround the spine.

Facet Joint – Joints in the spine that are on the either side of the centre of the spine and join 2 vertebrae.

Lumbar spine – the lower back

L5/S1 disc – the disc that is located between the 5th lumbar vertebra and the sacrum

Myotomes – groups of muscles that are controlled? by a specific nerve coming from the spine

Nerve root impingement – one of the nerves in the lower back is getting compressed by other structures (usually by the disc)

NSAIDs – non-steroidal anti-inflammatory drugs

Nucleus pulposis – the softer inner central portion of the disc

Radiculopathy – weakness, numbness and altered sensation in the leg.

Radicular pain – radiating pain in the leg that results from an irritated nerve

Somatic referred pain – pain from a tissue, like a muscle, ligament, or bone, that is felt somewhere else in the body. It usually feels dull, achy, and hard to localise.

Sacroiliac joint (SIJ) – a joint between the lower back (sacrum) and the pelvis.

Spinal stenosis – a condition where the spinal canal narrows and hence reduces the space that the nerves can travel through.

Spondylosis – umbrella term given to the general degeneration of the spine. These findings are commonly a normal part of ageing.

Spondylolisthesis – slippage of one of the vertebrae on the other.
