# Supplementary material for: A Decision Aid for Patients Considering Surgery for Sciatica: Codesign and User‐Testing With Patients and Clinicians
Source: Health Expect. 2024 Jun 19;27(3):e14111. doi: 10.1111/hex.14111 (PMC11186058; doi:10.1111/hex.14111)
Supplement: Supplementary file 4 — Appendix 4: Clinician user guide. [file HEX-27-e14111-s001.pdf]

DECISION AID FOR PATIENTS CONSIDERING SURGERY FOR SCIATICA

CLINICIAN USER GUIDE

1 WHAT IS A PATIENT DECISION AID?

A decision aid is a type of clinical tool that supports patients when there is **more than one treatment option** and:

Neither option is clearly better	OR	The options have benefits and harms that people value differently

"You can see that both are options. It all depends on the individual, their lives or lifestyle and what they want to do"

2 WHAT ARE THE TREATMENT OPTIONS IN THIS DECISION AID?

This decision aid helps patients with sciatica choose between:

1) Surgery

Going to hospital for surgery, along with rehabilitation exercises.

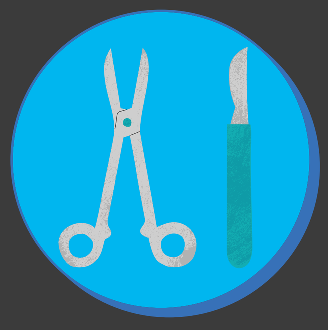

2) Try other options first

Delaying the decision to have surgery, to see if conservative management improves outcomes.

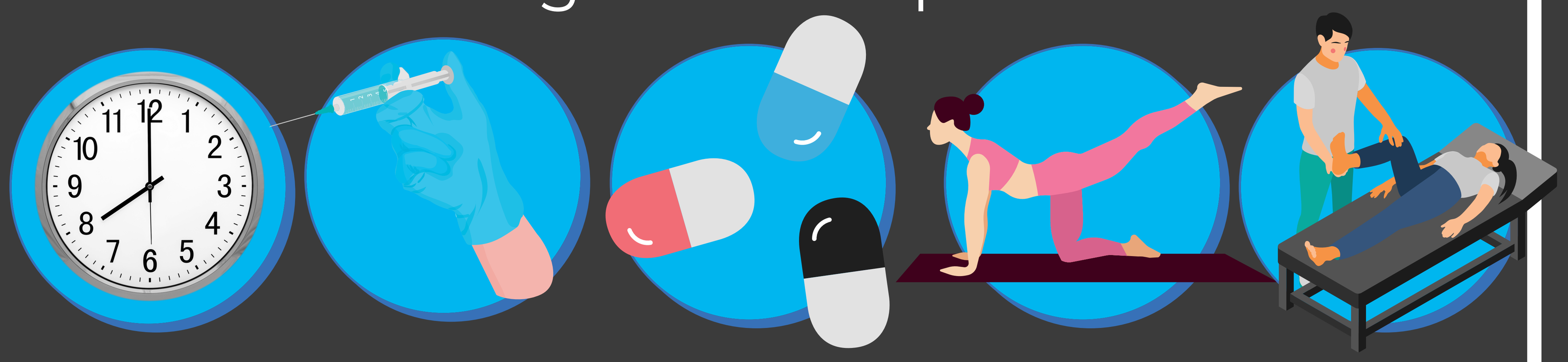

You will need to work with the patient to create an individualised plan.

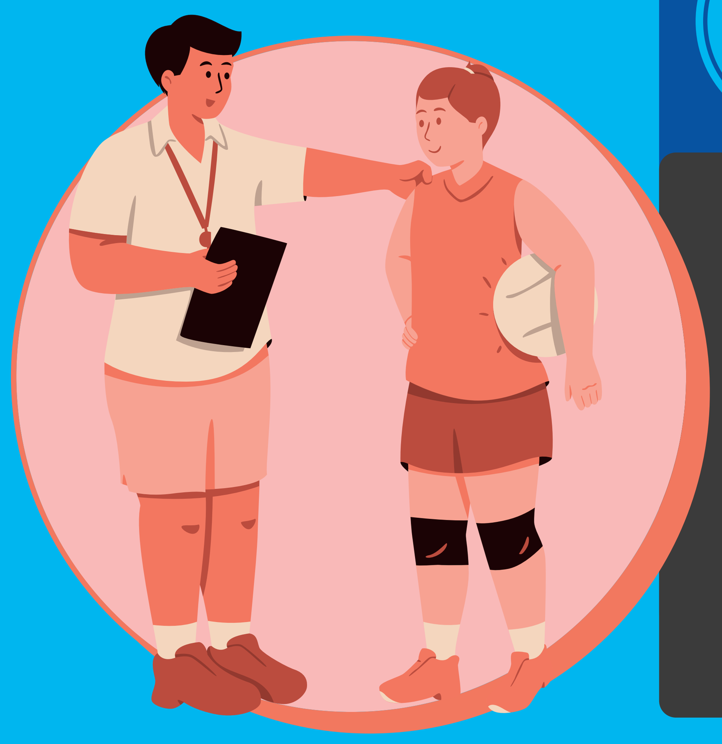

3 WHO IS IT MOST SUITABLE FOR?

The decision aid is more suitable for patients who:

- you consider appropriate for referral to a spinal surgeon for an opinion on back surgery, or patients who have already received a referral
- have sciatica likely caused by lumbar disc herniation
- have sciatic leg pain that has lasted less than 6 months (the evidence is less clear for longer durations) [1].

4 WHEN SHOULD I GIVE THE DECISION AID TO THE PATIENT?

This decision aid is designed for patients who have a referral to a surgeon.
You can give it to them **before** they see the surgeon.
The decision aid has three parts:

Before you visit the surgeon

- Learn about sciatica
- Learn about your options
- Think about what you need to ask the surgeon
- Think about what matters most to you.

During the visit to the surgeon

- The surgeon will tell you whether surgery is likely to help your sciatica
- Talk to the surgeon about what matters most to you
- Ask questions to make sure you understand your options.

After you visit the surgeon

- Consider your options
- Think about the evidence, what matters most to you, and the surgeon's advice.
- Make a decision about surgery.

5 WHAT DATA IS THE INFORMATION ABOUT TREATMENT OPTIONS BASED ON?

The key data in this decision aid comes from a landmark 2007 trial [2]. Four systematic reviews [3-6] identified this as the only high quality trial comparing sciatica to conservative management. Recommendations arising from the study are also generally consistent with those of other studies, including the latest systematic review (published 2023 [6]).

SINGLE SITE RANDOMISED CONTROLLED TRIAL

283 PATIENTS WITH LUMBAR SPINE CAUSES OF SCIATICA
(No more than grade 3 weakness)

The groups were randomised to:

SURGERY (N=141)
(microdiscectomy)

TRY OTHER OPTIONS FIRST (N=142)
(conservative management with opportunity for surgery at 6 months)

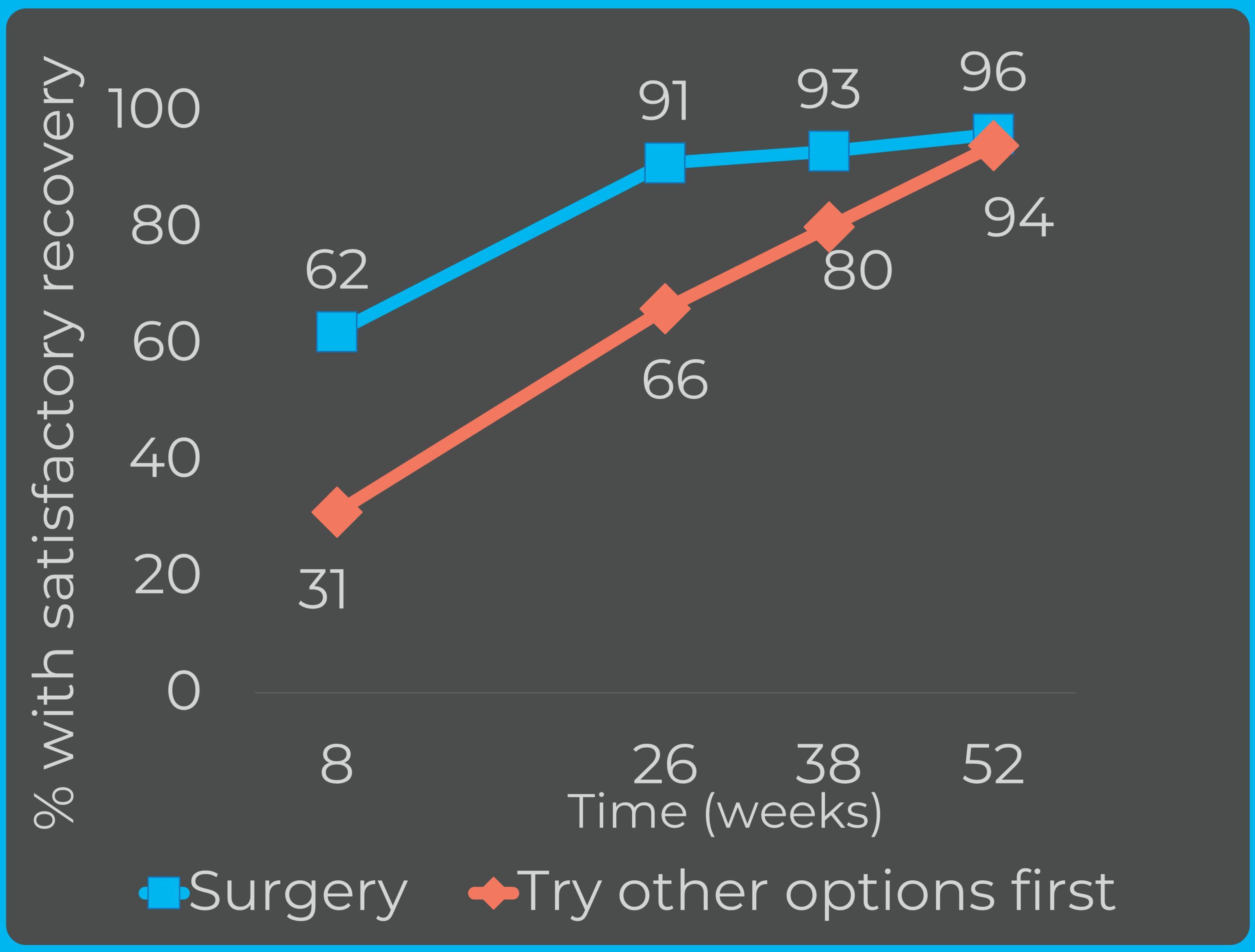

Early surgery showed significantly **faster recovery.**

However, by **1 year**, a similar proportion of participants in both groups had recovered.

Between 6 months and 1 year, 55 people in the "try other options first" group (39%) went on to have surgery.

*'Satisfactory recovery' reflects self-reported 'complete' or 'near complete' recovery on a 7-point Likert scale. This rating corresponds to a mean Roland Disability Questionnaire score of 1.3 (SD=2.3); mean leg pain score of 6.5 out of 100 (SD=13.7); mean back pain score of 10.2 out of 100 (SD=14.6).

6 WHAT ARE THE RELEVANT GUIDELINES?

Australian Commission for Safety and Quality in Health Care (Australia, 2022): https://www.safetyandquality.gov.au/standards/clinical-care-standards/low-back-pain-clinical-care-standard

National Institute for Health and Care Excellent (UK, last updated 2020): https://www.nice.org.uk/guidance/ng59

BMJ Clinical update (2019) https://www.bmj.com/content/367/bmj.l6273

"It's a good source of information that's not overwhelming. "

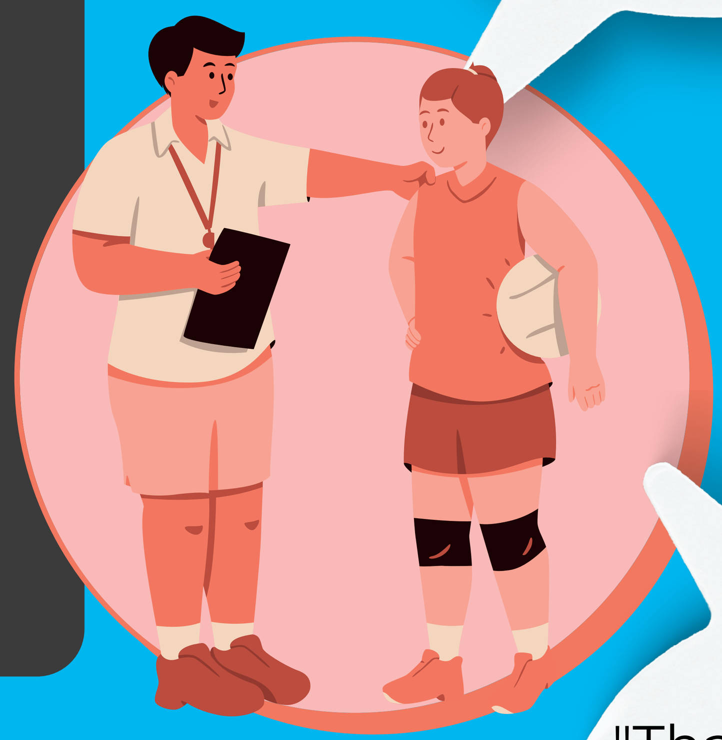

"That actually helped a lot in terms of putting into perspective what's important."

REFERENCES

[1] Bailey et al. (2020) “Surgery versus conservative care for persistent sciatica lasting 4 to 12 months,” NEJM, 382(12), DOI: 10.1056/nejmoa1912658.
[2] Peul et al. (2007) “Surgery versus prolonged conservative treatment for sciatica,” NEJM, 356(22), DOI:10.1056/nejmoa064039.
[3] Jacobs et al. (2011) Surgery versus conservative management of sciatica due to a lumbar herniated disc: a systematic review. European Spine Journal, 20(4), 513-522, DOI: 0.1007/s00586-010-1603-7
[4] Fernandez et al. (2016) Surgery or physical activity in the management of sciatica: a systematic review and meta-analysis. European Spine Journal, 25(11), 3495-3512. DOI: 10.1007/s00586-015-4148-y
[5] Clark et al. (2020) Surgical Management of Lumbar Radiculopathy: a Systematic Review. Journal of General Internal Medicine, 35(3), 855-864. DOI: 10.1007/s11606-019-05476-8
[6] Liu et al (2023). Surgical versus non-surgical treatment for sciatica: systematic review and meta-analysis of randomised controlled trials. BMJ, DOI: 10.1136/bmj-2022-070730

LINK TO PATIENT DECISION AID
